# The Role of the Multidisciplinary Team in the Management of Complex Cases of Liver Disease in a Peripheral Hospital: A Retrospective Analysis of MDT Registry

**DOI:** 10.1002/hsr2.71986

**Published:** 2026-03-18

**Authors:** Gabriele A. Vassallo, Tommaso Dionisi, Angelo Randazzo, Antonio Limblici, Monica Failla, Marcella Ferraro, Oriana Maiorana, Eleonora Taibi, Filomena Pietrantonio, Francesca Brovelli, Giovanni Addolorato, Giuseppe Augello

**Affiliations:** ^1^ Internal Medicine Unit Barone Lombardo Hospital Canicattì Agrigento Italy; ^2^ Internal Medicine and Alcohol Related Disease Unit, Columbus‐Gemelli Hospital Fondazione Policlinico Universitario A. Gemelli IRCCS Rome Italy; ^3^ Department of Medical and Surgical Sciences Università Cattolica di Roma Rome Italy; ^4^ Radiology Unit Barone Lombardo Hospital Canicattì Agrigento Italy; ^5^ Surgery Unit Barone Lombardo Hospital Canicattì Agrigento Italy; ^6^ Oncology Unit Barone Lombardo Hospital Canicattì Agrigento Italy; ^7^ Internal Medicine Unit, Medical Area Department Castelli Hospital Rome Italy

**Keywords:** diagnostic imaging, hospitals, community, interdisciplinary communication, liver diseases, patient care team, referral and consultation

## Abstract

**Background and Aims:**

In liver disease (LD), multidisciplinary teams (MDTs) support complex decision‐making and may improve care delivery. The present study aims to evaluate the role of a multidisciplinary approach in complex cases of LD in a peripheral hospital.

**Methods:**

We retrospectively analyzed a prospectively maintained MDT registry. Since November 2023, consecutive complex cases referred to the Liver Disease Outpatient Clinic of Barone Lombardo Hospital were discussed in MDT meetings. The initial proposals of clinicians were compared with the decisions taken by the team to evaluate the appropriateness of prescribing imaging tests and the need for referral. The MDT also re‐reviewed prior imaging and applied guideline‐concordant criteria to minimize low‐value testing.

**Results:**

Of 52 cases presented, 1 was excluded (non‐hepatic lesion), leaving 51 patients analyzed. The median age was 60 years, 32 patients (62.7%) were female, and 27 patients (52.9%) had chronic LD. A total of 22 imaging tests and 1 liver biopsy were avoided, and no patients were referred to a secondary or tertiary hospital.

**Conclusions:**

The multidisciplinary approach in a peripheral hospital appears to improve the appropriateness of prescribing imaging tests with consequent reduction of costs and contrast‐related adverse events. It also allows for a better allocation of resources and avoids unnecessary referrals to secondary and tertiary hospitals, with potential benefits for patient experience and diagnostic safety.

## Summary

- What's known
  ◦ Mcommunity /community hospitals across the broader LD spectrum is scarce.

- What's new
  ◦ In this real‐world series (*n* = 51), MDT review avoided 22 imaging tests and 1 biopsy and eliminated referrals to higher‐level hospitals.

- Clinical implications
  ◦ Implementing MDTs in community settings may curb low‐value imaging, reduce potential harm from contrast exposure, and optimize local resource use.

**Abbreviations**
ArLD: alcohol‐related liver disease
CT: computed tomography
LD: liver disease
MAFLD: metabolic dysfunction‐associated fatty liver disease
MDT: multidisciplinary team
MetALD: metabolic and alcohol‐related liver disease
MRI: magnetic resonance imaging

## 1 Introduction

Liver diseases (LD) represent a broad spectrum of conditions that can lead to severe health complications and high mortality rates [1]. As these conditions often involve complex and multiple pathophysiological processes, a multidisciplinary approach is essential for effective diagnosis, treatment, and management [2]. In practice, clinical complexity and decision uncertainty are frequent, especially when prior imaging or comorbidities obscure typical diagnostic pathways. The multidisciplinary approach involves healthcare professionals from various specialties working collaboratively to provide holistic care for patients. In the context of LD, a multidisciplinary team (MDT) typically includes hepatologists, surgeons, radiologists, and pathologists [3] and, where appropriate, oncologists and endoscopists to address oncology‐ and procedure‐related scenarios. Each specialist brings unique expertise, allowing for a more effective approach to patient care. MDTs are successfully used for the management of hepatocellular carcinoma and evaluating candidacy for liver transplantation [2, 4]. A single‐center study, enrolling patients admitted with liver cirrhosis, showed that specialized post‐discharge multidisciplinary outpatient care is associated with decreased acute care utilization and improved 1‐year transplant‐free survival [5]. However, robust real‐world evidence on MDTs across the broader spectrum of LD in peripheral hospitals is limited [6]. In these settings, constrained resources and limited sub‐specialty access may translate into delayed diagnosis, duplicated or low‐value imaging, avoidable referrals, and potential contrast‐related risks [7, 8].

This paper explores the pivotal role of a MDT in enhancing patient outcomes, streamlining care processes, and ensuring comprehensive management of LD in these healthcare settings. Specifically, we aimed to quantify: (i) the proportion of initially proposed imaging judged inappropriate versus appropriate after MDT review, (ii) the number and type of imaging tests and biopsies avoided, (iii) the rate of referrals to secondary/tertiary centers, and (iv) safety during follow‐up (liver‐related complications and all‐cause mortality). These measurable endpoints reflect resource use (tests avoided), patient experience (referrals avoided), and diagnostic safety.

## 2 Materials and Methods

This was a retrospective observational analysis of a prospectively maintained MDT registry. Since November 2023, consecutive complex cases of LD referred to the Liver Disease Outpatient Clinic, Surgery and Oncology Units of Barone Lombardo Hospital were discussed in MDT meetings by a team consisting of hepatologists, radiologists, oncologists, surgeons, and an endoscopist. The hepatologists involved in the team were board‐certified in General Internal Medicine and had extensive experience in treating LD.

In the present study, complex LD was defined as cases of uncertain diagnosis, overlap conditions, or necessitating evaluation from several specialties to delineate an appropriate management strategy. These include patients with focal liver lesions not clearly delineated on initial imaging, patients with complications of the liver in the context of surgical or oncological disease, and patients in whom malignancies of the liver or pathologies of the biliary tree were envisaged but not delineated. Whether a case was presented to the multidisciplinary meeting was determined by an apparent need for collective expertise in management, treatment, or follow‐up approach.

The MDT met monthly on the first Thursday of the month, and the cases collected in the previous weeks were discussed. All patients provided written informed consent before MDT; institutional approval/waiver details are reported in the Declarations.

The meeting took place at the Radiology Unit, where imaging was jointly examined and reviewed. After the meeting, the decision taken by the team was communicated to the patient, and all team members applied the agreed clinical pathways. Cases of children and pregnant women were not taken into consideration due to the lack of pediatricians and gynecologists, respectively, in the team.

In total, 52 cases were discussed in MDT. One case was excluded from the statistical analysis because, although the patient had been referred to the Liver Disease Outpatient Clinic for metabolic dysfunction‐associated fatty liver disease (MAFLD), the discussion regarded the finding in the abdominal computed tomography (CT) of a focal pancreatic lesion. In this study, the initial proposals of clinicians, to whom the cases had initially been addressed, were compared with the final decisions taken by the team. Recording of the initial proposal and data sources (bias minimization). For each case, the referring clinician's initial diagnostic and/or treatment plan was captured before the MDT discussion, recorded verbatim from the referral letter and/or the electronic health record (EHR) order/request. A standardized pre‐MDT proforma was used to abstract data. When both sources were available, two investigators reconciled discrepancies prior to the meeting; unclear entries were coded as “unclear initial proposal.” All timestamps documented the pre‐MDT timing to reduce hindsight bias.

For each case, the initial diagnostic and treatment plan proposed by the referring clinician was recorded before the MDT discussion. After the MDT meeting, the final consensus decision was documented on the same proforma and compared with the initial decision of the referring clinician to assess changes in radiological imaging recommendations (type and necessity of further radiological investigation), treatment plans, and need for referral to a secondary or tertiary hospital.

The main outcomes are the number of avoided imaging tests because they were considered unnecessary/inappropriate (not aligned with current clinical guidelines/recommendations) and the number of patients referred to higher‐level hospitals. The secondary outcome is the number of liver‐related complications or death during follow‐up.

Appropriateness of imaging tests was determined by multidisciplinary discussion. An imaging test was deemed inappropriate if it did not comply with clinical guidelines released by the “American Association for the Study of Liver Diseases” and the “European Association for the Study of the Liver,” or if it was a repeat of recent tests without an obvious clinical indication [9, 10, 11]. Conversely, imaging was appropriate if it was directly contributing to diagnostic resolution, staging, or guiding therapy planning. All patient history available at that point was reviewed, together with previous imaging tests, by the reviewing MDT. Depending on this review, an output metric was recorded on the number of imaging tests avoided—“avoided imaging tests.”

The study was conducted in accordance with the Declaration of Helsinki and current Italian data‐protection legislation; all participants provided written informed consent.

The design, conduct, and reporting of this study adhered to the STROBE (Strengthening the Reporting of Observational Studies in Epidemiology) guidelines [12, 13].

## 3 Statistical Analysis

Data were analyzed using Stata 18. Descriptive statistics were computed for demographic and clinical characteristics of the study population. Continuous variables were expressed as means and standard deviations or medians with interquartile ranges, depending on data distribution assessed by the Shapiro–Wilk test. Categorical variables were summarized as frequencies and percentages. No formal hypothesis testing was performed; analyses were descriptive. For key proportions, 95% confidence intervals were calculated to indicate precision [14, 15]. No a priori sample‐size calculation was performed; the sample size was defined by the number of consecutive cases recorded in the MDT registry during the study period.

## 4 Results

Cohort characteristics. In total, 51 cases were included. The median age was 60 years, 32 patients (62.7%) were female, and 19 (37.3%) were male. Among all patients, 27 (52.9%) had chronic LD; specifically, 16 patients were affected by advanced LD and 11 by early LD. Among patients with LD, the etiology was (33.3%) MAFLD, (25.9%) alcohol‐related liver disease (ArLD), (14.8%) metabolic and alcohol‐related liver disease (MetALD), (11.1%) chronic viral hepatitis, and (11.1%) cryptogenic. Overall, 39 (76.4%) were discussed for diagnostic purposes, 16 (31.3%) for therapeutic decisions, and 4 (7.8%) for both. Thirty‐five cases were discussed for focal liver lesions (6 cystic and 29 solid) and 9 for biliary tree disease. The general characteristics and the type of lesion/underlying LD of cases discussed by the MDT are shown in Tables 1 and 2.

**Table 1:** General characteristics of the patients.

General characteristics of patients	
Age (yr), median (range)	60 (16–84)
Gender	
Male, *n* (%)	19 (37.3)
Female, *n* (%)	32 (62.7)
Liver disease patients *n* (%)	27 (52.9)
Early liver disease *n* (%)	11 (21.5)
Advanced liver disease *n* (%)	16 (31.4)
Etiology of liver disease	
Metabolic dysfunction‐associated steatotic liver disease (MASLD), *n* (%)	9 (33.3)
Alcohol‐related liver disease (ArLD), *n* (%)	7 (25.9)
Metabolic and alcohol‐related liver disease (MetALD)	4 (14.8)
Chronic viral hepatitis, *n* (%)	4 (14.8)
Cryptogenetic liver disease, *n* (%)	3 (11.1)

Abbreviations: *n*, number of patients; yr, years.

**Table 2:** Lesion or underlying liver disease of patients discussed by the MDT.

Liver cancer	
Hepatocellular carcinoma, *n*	10
Liver metastasis, *n*	4
Benign focal liver lesions	
Hemangiomas, *n*	4
Regenerative liver nodule, *n*	4
Focal nodular hyperplasia (FNH), *n*	2
Cystic lesion, *n*	2
Solitary necrotic nodules, *n*	2
Polycystic liver disease (PLD), *n*	1
Focal hepatic steatosis, *n*	1
Hepatic infectious lesions	
Suspected relapse of echinococcosis, *n*	4
Acute/chronic liver disease	
Drug‐induced liver injury, *n*	3
Wilson's disease, *n*	1
Liver transplantation, *n*	2
Steatotic liver disease, *n*	1
Cancer of biliary tract	
Cholangiocarcinoma, *n*	3
Benign biliary tract disease	
Adenomyomatosis, *n*	1
Gallbladder polyp, *n*	1
Choledochal cyst, *n*	1
Cholelithiasis, *n*	1
Intracholecystic papillary neoplasm, *n*	1
Suspected primary biliary cholangitis, *n*	2

Abbreviation: *n*, number of patients.

Diagnostic cases and imaging appropriateness. Among cases discussed for diagnostic purposes (39), in 11 (28.2%) cases, the initially prescribed imaging tests were considered inappropriate by the MDT, while in 7 (17.9%), they were considered appropriate. In 11 (28.2%) cases, no further imaging or follow‐up was planned by the MDT, while in 10 (25.7%) cases, prior imaging tests were reviewed and discussed in the MDT.

Resource use and planned follow‐up. A total of 3 abdominal ultrasounds, 3 abdominal CT scans, 15 upper abdominal magnetic resonance imaging (MRIs) examinations, 1 positron emission tomography (PET) scan, and 1 liver biopsy were avoided as determined by the MDT versus the individual physician's initial assessment. A radiological follow‐up or further investigation with a more appropriate imaging test was planned for 22 (56.4%) patients: follow‐up ultrasound in 16 cases, further investigation with CT in 2, with MRI in 2, and with liver biopsy in 2. No further follow‐up or further investigation was planned in the remaining 17 (43.6%) cases.

Referrals and safety outcomes. No patients were referred to a secondary or tertiary hospital for consultation. The mean follow‐up period was 216 days (17–437). No adverse events were reported during the follow‐up period except for a death (1.9%), not related to the underlying LD. The study's results are summarized in Table 3.

**Table 3:** The main results of the study.

Results	
Radiological exams considered inappropriate, *n* (%)	11 (28.2%)
Radiological exam considered appropriate, *n* (%)	7 (17.9%)
No further investigation or follow‐up, *n* (%)	11 (28.2%)
Saved radiological exams, *n* (%)	22 (56.4%)
Abdominal ultrasounds, *n*	3
Abdominal CT scan, *n*	3
Abdominal MRI, *n* (%)	15
Positron emission tomography scans, *n*	1
Saved liver biopsy, *n* (%)	1 (100%)
Patients referred to higher level hospitals, *n*	0
Secondary hospitals, *n*	0
Tertiary hospitals, *n*	0
Adverse events	
Death	1 (1.9%)
Liver‐related death	0
Length of follow‐up (days), mean (range)	216 (17–437)

Abbreviation: *n*, number of patients.

## 5 Discussion

The multidisciplinary approach improves the appropriateness of prescribing imaging tests with consequent better allocation of resources, reduction of costs, and fewer contrast‐related adverse events [16]. Moreover, the MDT avoids unnecessary referrals to secondary and tertiary hospitals. Based on our retrospective analysis of a prospectively maintained MDT registry, these effects likely stem from two complementary mechanisms: structured re‐review of prior imaging and clinical data and the application of guideline‐concordant criteria that curb low‐value testing and streamline decisions. These mechanisms may also enhance patient experience (fewer trips and waiting times) and diagnostic safety (fewer incidental cascades), particularly in peripheral settings [17].

The management of complex LD exemplifies the importance of a multidisciplinary approach. Each specialty brings unique expertise and perspectives, contributing to a comprehensive and cohesive treatment plan. The interaction between various disciplines can enhance diagnostic accuracy, optimize therapeutic strategies, and improve patient outcomes [3]. Hepatologists play a central role in the medical management of LD. They are responsible for interpreting liver function tests, planning the treatment, and managing complications. Their expertise is crucial in determining the need for liver transplantation and the management of liver cancer [2]. Radiologists contribute significantly through imaging techniques such as ultrasound, CT scans, and MRI, which are essential for accurate diagnosis and monitoring of liver conditions [18]. Oncologists specialized in hepatocellular carcinoma and other liver malignancies collaborate with hepatologists, radiologists, and surgeons to provide comprehensive cancer care [18]. Surgeons are indispensable in the treatment of LD requiring surgical intervention; endoscopists, as well, are vital for diagnosing and treating complications related to LD, such as variceal bleeding [19]. Beyond role descriptions, the MDT format reduces uncertainty at key decision points (e.g., indeterminate focal lesions), aligning test selection with pre‐test probability and clinical utility.

By leveraging the diverse expertise within the team and coordinating healthcare providers, the MDT might optimize the use of available resources, especially in places with limited access to advanced technologies and specialists. Moreover, the integration of care pathways and personalized treatment plans are critical components of successful multidisciplinary management. Our findings (tests and one biopsy avoided; zero inter‐hospital referrals) are consistent with this optimization, suggesting a potential budget impact in addition to clinical benefits.

The present study's findings align with previous studies enrolling patients with LD. Addolorato and colleagues reported the role of the MDT in managing ArLD patients before and after liver transplantation in terms of reduction of relapse and mortality after the liver transplantation [20, 21, 22]. Similarly, Targher et al. highlighted the need for a multidisciplinary approach for managing patients with MAFLD to deliver holistic and person‐centered care [23]. However, unlike work focused primarily on hepatocellular carcinoma or transplant pathways [2], the results of this study indicate that MDTs are also valuable in a wider range of liver conditions, such as benign focal lesions, biliary disease, and other hepatic diseases. Each of these professions brought their own area of expertise to case review, particularly for indeterminate or borderline cases. This breadth reflects routine case‐mix in peripheral hospitals and underscores the external relevance of MDTs beyond tertiary centers.

Whereas earlier literature focused on the theoretical advantage of MDTs, these findings present real‐world evidence that these MDTs can decrease low‐value care and improve diagnostic accuracy in non‐tertiary care settings [4, 7, 8]. In addition, fewer referrals may mitigate travel/time burden and fragmentation of care, outcomes valued by patients and systems alike. Future comparative studies should quantify these patient‐reported experience measures (PREMs) and model cost‐effectiveness using local tariffs [24, 25].

Finally, peripheral hospitals often face limitations such as fewer specialists, reduced access to advanced diagnostic tools, and financial constraints [6]. These challenges can impede the effective management of complex LD cases. However, the integration of MDTs can mitigate these issues by pooling expertise and optimizing resource use [3]. Our data support this mitigation, showing targeted use of imaging and consensus‐based pathways without recourse to higher‐level referral.

### 5.1 Future Perspectives

The incorporation of MDTs for the management of complex LD cases in peripheral hospitals is transformative. It bridges the gap between limited resources and the need for specialized care, ultimately leading to improved patient outcomes and more efficient healthcare delivery. Through regular team meetings and coordinated care plans, a peripheral hospital could successfully manage several complex cases that would otherwise require referral to distant, specialized centers, also causing discomfort for patients. Moreover, efficient resource utilization can lead to cost savings and a better allocation of hospital funds. Next steps include: (i) prospective comparative designs with explicit economic endpoints (budget impact/cost‐effectiveness), (ii) systematic capture of PREMs to quantify patient experience, and (iii) safety dashboards tracking unplanned imaging, ED visits, and delayed diagnoses.

Based on these preliminary observations, future prospective multicenter studies using a validated and standardized method are needed to confirm this clinical approach in the management of complex cases of LD in peripheral hospitals.

### 5.2 Limits

This study has some limitations. Firstly, this is a retrospective observational analysis of a prospectively maintained MDT registry without a control group, which limits causal inference; secondly, the small sample size and the monocentric design. Moreover, selection bias could also be present in the study.

Moreover, the cohort is small and monocentric, and the sample size was not determined a priori (we included all consecutive MDT cases during the study period); therefore, estimates have limited precision, and the study may be underpowered to detect infrequent outcomes.

Misclassification of the initial proposal cannot be fully excluded despite standardized abstraction; follow‐up duration was relatively short and variable.

The lack within the MDT of pathologists who can interpret and reanalyze liver biopsies to provide a more accurate diagnosis is another limitation of the study. This will be addressed in future iterations by including a pathology representative for select cases.

## 6 Conclusion

In conclusion, deploying MDTs in peripheral hospitals is a strategic approach that can significantly enhance the management of complex LD cases, offering a model for a better healthcare delivery in challenging settings. The collaboration among hepatologists, radiologists, surgeons, and oncologists ensures a more comprehensive and holistic care for the patients. Our results suggest reduced low‐value imaging, avoidance of invasive procedures, and elimination of inter‐hospital referrals over the observation window. Future research should focus on refining these integrated care models and exploring the impact of multidisciplinary interventions on long‐term patient outcomes as well as economic and patient‐experience endpoints.

## Author Contributions

**Gabriele A. Vassallo:** conceptualization, methodology, investigation, data curation, formal analysis, writing – original draft. **Tommaso Dionisi:** conceptualization, methodology, investigation, data curation, formal analysis, writing – original draft. **Angelo Randazzo:** investigation, writing – original draft. **Antonio Limblici:** methodology. **Monica Failla:** investigation. **Marcella Ferraro:** investigation, data curation. **Oriana Maiorana:** investigation, methodology. **Eleonora Taibi:** investigation, data curation. **Filomena Pietrantonio:** writing – review and editing. **Francesca Brovelli:** formal analysis, visualization. **Giovanni Addolorato:** conceptualization, writing – review and editing, supervision, project administration. **Giuseppe Augello:** conceptualization, writing – review and editing, supervision, project administration. All authors have read and approved the final version of the manuscript.

## Funding

The authors received no specific funding for this work.

## Ethics Statement

This retrospective, non‐interventional observational study was carried out in accordance with the Declaration of Helsinki (2013 revision); Regulation (EU) 2016/679 (General Data Protection Regulation, GDPR); and the Italian Data Protection Code (Legislative Decree 196/2003) as amended by Legislative Decree 101/2018, specifically Art. 110‐bis governing scientific research. All participants provided written informed consent for the collection and research use of their de‐identified clinical data before enrollment.

## Conflicts of Interest

The authors declare no conflicts of interest.

## Transparency Statement

The lead author Tommaso Dionisi affirms that this manuscript is an honest, accurate, and transparent account of the study being reported; that no important aspects of the study have been omitted; and that any discrepancies from the study as planned (and, if relevant, registered) have been explained.

## Data Availability

De‐identified data that support the findings of this study are available from the corresponding author upon reasonable request.
